# The eminent roles of ncRNAs in the pathogenesis of psoriasis

**DOI:** 10.1016/j.ncrna.2020.06.002

**Published:** 2020-07-03

**Authors:** Soudeh Ghafouri-Fard, Reyhane Eghtedarian, Mohammad Taheri, Azadeh Rakhshan

**Affiliations:** a Department of Medical Genetics, Shahid Beheshti University of Medical Sciences, Tehran, Iran; b Urogenital Stem Cell Research Center, Shahid Beheshti University of Medical Sciences, Tehran, Iran; c Department of Pathology, Shahid Beheshti University of Medical Sciences, Tehran, Iran

**Keywords:** miRNA, lncRNA, Psoriasis

## Abstract

Psoriasis is a chronic immune-related disorder in which both genetic and environmental parameters are involved. Recent studies have demonstrated dysregulation of long non-coding RNAs (lncRNAs) and microRNAs (miRNAs) in the peripheral blood or skin lesions of patients with psoriasis. While a number of lncRNAs such as MEG3, AL162231.4 and NONHSAT044111 have been down-regulated in the course of psoriasis, others including PRINS, MIR31HG, RP6‐65G23.1, MSX2P1, SLC6A14-1:1, NR_003062 have been up-regulated. Moreover, expressions of several miRNAs have been dysregulated in this disorder. Among dysregulated miRNAs are miR-126, miR-143, miR-19a and miR-155 whose diagnostic roles in the psoriasis have also been assessed. Dysregulated non-coding RNAs in this disorder participate in the regulation of chemokine signaling pathway and immune response, control of epidermal development and skin barrier as well as modulation of function of certain subsets of T cells. Besides, these transcripts possibly regulate activity of NF-κΒ, mTOR, MAPK and JAK-STAT signaling pathways. Besides, expression levels of circRNAs have been decreased in the psoriasis lesions. Massive alterations in the levels of lncRNAs and miRNAs in the psoriasis lesions or peripheral blood of affected individuals show participation of these transcripts in the pathogenesis of this disorder.

## 1 Introduction

Psoriasis is a chronic immune-related disorder of skin which is caused by complex interactions between genetic and environmental parameters [1]. Several lines of evidence suggest the role of activated T cells in the pathogenesis of psoriasis. First, activated CD4^+^ and CD8^+^ T cells have been detected in the psoriatic lesions and blood of affected patients [2,3]. Moreover, clonally expanded T cell receptor β-chain rearrangements have been observed in the plaques of psoriasis patients. This antigen-specific T cell selection shows the persistent pathogenic T cell response in these patients [4]. Finally, investigations in the xenograft models of psoriasis have indicated the significance resident T cells in the evolution of psoriasis and their role in the production of TNF-α in these lesions [5]. Based on the role of immunological factors in the development of psoriasis, therapeutic strategies against TNF-α, IL-23, and IL-17 have been applied in this disorder [6]. A number of factors such as mechanical stress, seasonal elements, infectious factors, ultraviolent radiation, and a number of medications such as β-blockers have been recognized as extrinsic risk factors for psoriasis [6]. Moreover, some disorders such as hypertension, diabetes mellitus and cardiovascular disorders have been demonstrated to co-occur with psoriasis [6]. Recent studies have indicated the role of non-coding RNAs (ncRNAs) in the pathogenesis of psoriasis [7]. Being classified based on their sizes into long noncoding RNAs (lncRNAs) and microRNAs (miRNAs), these transcripts are involved in the regulation of immune responses at several regulatory levels [8,9]. MiRNAs mostly regulate immune responses at the translation level. A number of miRNAs namely the miR-17–92 cluster, miR-150, miR-155, miR-181 and miR-223 are abundantly expressed in the immune cells and participate in the maturation, differentiation and function of these cells [9]. Notably, several of these transcripts have been dysregulated in psoriasis plaques or peripheral blood of patients with this skin disorder [10,11]. As regulators of gene expression at transcriptional and post-transcriptional levels, lncRNAs participate in the preservation of hematopoietic stems cells, the differentiation and survival of myeloid cell and the activity of several effector cells in the immune system [8]. In the current manuscript, we review and summarize the role of ncRNAs in the pathogenesis of psoriasis.

## 2 LncRNAs and psoriasis

A recent microarray analysis lncRNAs profiles in psoriatic skin samples and normal skin samples have shown dysregulation of more than 2000 lncRNAs most of them having *cis*- or *trans*-regulated predicted target genes. Dysregulated lncRNAs were enriched in signaling pathways that control immune responses such as JAK-STAT, Wnt and Toll-like receptor as well as cytokine and chemokine related pathways [12]. Another high throughput data analysis revealed reported differential expression of hundreds of lncRNAs between psoriasis lesions and uninvolved skin samples from these patients or normal skin samples. Differentially expressed lncRNAs were enriched in immune related pathways and those participate in the epidermal differentiation [13]. Jia et al. have made an in vitro model for assessment of the role of MEG3 in the pathogenesis of using HaCaT and HHEK cells. Expression of this lncRNA was decreased in these cells and in psoriatic skin specimens. MEG3 has been shown to suppress proliferation and enhance apoptosis of activated cells through modulation of miR-21 which inhibit caspase-8 expression [14]. Li et al. assessed RNA-Seq data of psoriatic and normal skin samples to recognize psoriasis-related lncRNAs and mRNAs. They reported a number of differentially expressed lncRNAs and mRNAs and constructed the interaction network between them. Finally, they validated expression of an mRNA/lncRNA pair (CCL27 and AL162231.4) in psoriatic and normal skin samples. Based on their results, expression of both genes were down-regulated in the affected samples [15]. Table 1 shows the list of down-regulated lncRNAs in the psoriasis.

**Table 1:** List of LncRNAs whose expression has been down-regulated in the psoriasis.

LncRNA	Numbers of clinical samples	Source	Targets/Regulators	Signaling Pathways	Function and comments	Reference
MEG3	19 cases and 19 controls were recruited in this study.	HaCaT, HHEKs and psoriatic skin samples	Caspase-8, cytc, apaf-1 and miR-21	MEG3/miR-21 signaling	LncRNA MEG3 affects the proliferation and apoptosis through regulating miR-21 expression and subsequently caspase-8.	[14]
AL162231.4	30 patients with psoriasis and 28 healthy controls were recruited in this study.	Skin samples	CCL27	Chemokine signaling pathway, the immune response, epidermal development and skin barrier	The downregulation of LncRNA-AL162231.4 regulates CCL27 expression.	[15]
NONHSAT044111	15 patients with psoriasis and 15 healthy controls were recruited.	Skin samples		Differentiation and function of Treg cells, NF-κΒ, mTOR, MAPK and JAK-STAT signaling pathway and the release of cytokines and chemokines	The mentioned lncRNAs may be used as potential biomarkers and therapeutic targets for the treatment of psoriasis.	[12]
PPM1N-1:1						
GRHL2-11:1						
JAKMIP2-1:1						
GGTLC1-2:1						
NONHSAT025181						
ENST00000447257						
ENST00000623414						
ENST00000415656						
LINC00273-22:1						

PRINS is an up-regulated lncRNA in the affected and non-affected psoriatic epidermis whose expression is activated by stress. Szegedi et al. have demonstrated that PRINS silencing has changed cell morphology and gene expression profile. PRINS regulates the anti-apoptotic gene G1P3 in keratinocytes. G1P3 is remarkably over-expressed in hyperproliferative affected and non-affected epidermal cells of psoriatic patients compared with normal epidermis. Their experiments demonstrated that dysregulation of the PRINS might diminish sensitivity of keratinocytes to spontaneous apoptosis through modulation of G1P3 [16]. Gao et al. have reported up-regulation of MIR31HG in the affected psoriatic skin compared with normal skin. MIR31HG silencing has suppressed proliferation of keratinocytes and prompted cell cycle arrest in the G2/M phase. Up-regulation of MIR31HG has been shown to be associated with NF-κB [17]. Table 2 shows the list of lncRNAs whose expression has been up-regulated in the psoriasis.

**Table 2:** List of lncRNAs whose expression has been up-regulated in the psoriasis.

LncRNA	Numbers of clinical samples	Source	Targets/Regulators	Signaling Pathways	Function and comments	Reference
PRINS	6 mm punch biopsies were taken from non-lesional and lesional (n = 4, each) skin areas of patients with psoriasis. Skin biopsies obtained from healthy individuals (n = 7) undergoing plastic surgery were used as control samples.	Keratinocytes	G1P3	Apoptosis	G1P3 suppresses spontaneous keratinocyte apoptosis and its high expression in psoriatic skin participates to increased cell production rate and epidermal thickness in psoriasis. Overexpression of PRINS in Psoriatic non-lesional epidermis may act as a trigger factor to induce the expression of the anti-apoptotic G1P3 protein that participates to the maintenance of the keratinocyte hyperproliferation in the evolving psoriatic lesions.	[16]
MIR31HG	Punch biopsies (4 mm) were taken from lesional skin areas of 10 patients with psoriasis. Normal skin specimens were taken from healthy individuals undergoing plastic surgery.	HaCaT keratinocytes/Lesional skin tissues	IL-17A, IL-22, TNF-α or IL-1α target NF-κB signaling. MIR31HG expression is dependent on NF-κB activation.	NF-κB signaling	siRNA-mediated MIR31HG depletion induced cell cycle arrest in the G2/M phase. MIR31HG expression depends on NF-κB activation.	[17]
RP6‐65G23.1	HaCaT cell, a human skin epithelial cell line, and Human Normal Primary Epidermal Keratinocytes (HEKn) were used. M5, a cocktail of cytokines, was used to induce psoriatic inflammation like condition in HaCaT cells and primary keratinocytes.	HaCaT and HEKn cell lines	p21, p27, Bcl‐xl and Bcl2	P-ERK1/2/p‐AKT signaling pathway	Knockdown of RP6‐65G23.1 resulted in defects of growth and increased rates of apoptosis in HaCaT cells.	[18]
MSX2P1	10 cases of psoriatic lesions and 10 cases of normal skin tissues were collected.	Keratinocytes, HaCaT and HNEK cells	miR-6731-5p and S100A7		MSX2P1 acts as an endogenous sponge directly binding to miR-6731-5p and thus, resulting in elevated levels of S100A7 and other proinflammatory cytokines, by suppressing the expression of miR-6731-5p and inducing apoptosis in IL-22-stimulated keratinocytes.	[19]
SLC6A14-1:1	15 patients with psoriasis and 15 healthy controls were recruited.	Skin samples		Differentiation and function of Treg cells, NF-κΒ, mTOR, MAPK and JAK-STAT signaling pathway and the release of cytokines and chemokines	The mentioned lncRNAs may be used as potential biomarkers and therapeutic targets for the treatment of psoriasis.	[12]
NR_003062						
SERPINB3-4:1						
NONHSAT006518						
IGFL3-6:1						
ENST00000472053						
NONHSAT006509						
NR_030617						
RAPGEF2-3:1						
RPP40-3:3						

Few studies have appraised associations between single nucleotide polymorphisms (SNPs) within lncRNAs and risk of psoriasis. Rakhshan et al. have genotyped four ANRIL SNPs (rs1333045, rs1333048, rs4977574 and rs10757278) in a population of Iranian patients with psoriasis and healthy controls. They reported associations between the C allele of rs1333048 and the G allele of the rs10757278 and risk of psoriasis, while the A allele of the rs4977574 was recognized as protective allele. Certain ANRIL haplotypes were also associated with risk of this disorder in Iranian population [20]. The same group has also assessed the association between the rs12826786, rs1899663 and rs4759314 SNPs of the HOTAIR. They reported association between the rs12826786 and psoriasis in a way that T allele of this SNP conferred risk of psoriasis. They reported no significant difference in HOTAIR haplotype frequencies between patients and healthy subjects [21]. Table 3 summarizes the results of studies which assessed association between lncRNAs variants and risk of pasoriasis.

**Table 3:** Variants within lncRNAs which are associated with risk of psoriasis.

LncRNA	Numbers of clinical samples (tissues, serum, etc.)	Risk variant	Reference
*ANRIL*	286 patients with psoriasis and 300 age-/sex-matched controls were recruited.	rs1333048	[20]
		rs10757278	
		rs4977574	
*HOTAIR*	286 patients with psoriasis and 300 age-/sex-matched controls were recruited.	rs12826786	[21]

## 3 miRNAs and psoriasis

**Fig. 1:**
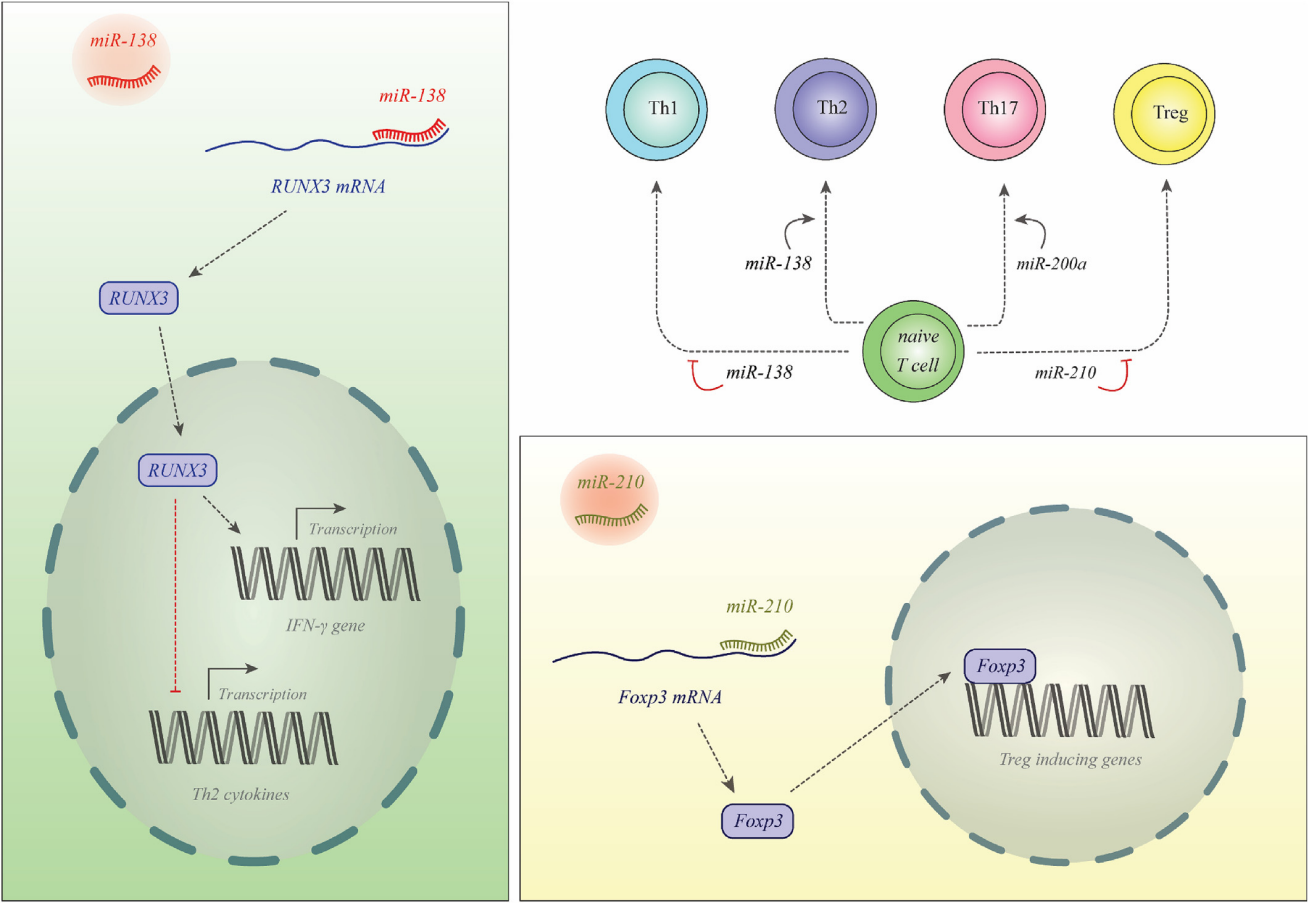
miR-138 is decreased in patients with psoriasis. This miRNA binds with 3′ UTR of RUNX3 to suppress its expression. Thus, down-regulation of miR-138 leads to up-regulation of RUNX3 which increases Th1/Th2 ratio [23]. miR-210 is increases in the psoriasis. This miRNA binds with 3′ UTR of FOXP3. FOXP3 is a master transcription factor for differentiation of Treg cells. Thus, miR-210 over-expression leads to reduction in the numbers of Treg cells [24].

These small ncRNAs are originated from larger primary RNAs and are processed into approximately 22 nucleotide transcripts. These mature transcripts regulate expression of their target genes at post-transcriptional level. Dysregulation in the expression of these transcripts contribute in the pathogenesis of psoriasis [22]. Fig. 1 shows the mechanism of involvement of two miRNAs in the pathogenesis of psoriasis.

## 4 Down-regulated miRNAs in psoriasis

Xu et al. demonstrated down-regulation of miR-125b in the psoriasis. The main cell type accountable for down-regulation of miR-125b levels in psoriasis lesions has been the keratinocyte. Overexpression of this miRNA in primary human keratinocytes inhibited proliferation and increased transcription of numerous differentiation markers. Functional studies revealed interaction between miR-125b and FGFR2 [25]. This miRNA has also been shown to interact with BRD4 and inhibit expression of the Jagged-1 ligand. Moreover, miR-125b suppresses Notch signaling pathway and inhibit the proliferation of psoriasis cells [26]. Let-7b has also been down-regulated in the psoriasis. Over-expression of this miRNA in the keratinocytes suppresses acanthosis and decreases the disease severity following treatment with imiquimod. Let-7b enhances differentiation of keratinocytes through suppression of IL-6 expression. This miRNA also negatively modulates the ERK signaling pathway in the keratinocytes of patients with psoriasis [27]. Another study has assessed expression of miRNAs in the keratinocytes of patients with psoriasis and control samples using the miRNA microarray technique and subsequent real time PCR method. Authors have demonstrated down-regulation of miR-145-5p in psoriatic skin lesions. Functional studies have verified the inhibitory effects of this miRNA on MLK3 expression. Moreover, up-regulation of miR-145-5p in normal human epidermal keratinocytes has been shown to inhibit cell proliferation and chemokine production. This miRNA controls activity of NF-κB and STAT3 signaling through modulation of MLK3. Notably, transfer of agomiR-145-5p into the skin cells has reduced epidermal hyperplasia and has amended the psoriasis-associated phenotypes [28]. Moreover, expression of miR-187 has been decreased in inflammatory cytokines-induced keratinocytes and skin samples obtained from patients with psoriasis. This miRNA suppresses keratinocytes hyperproliferation through inhibiting CD276 expression. Experiments in animal model of psoriasis have shown the role of this miRNA in reducing acanthosis and decreasing the severity of psoriasis [29]. miR-194 is another downregulated miRNA in skin lesions of patients with psoriasis. Up-regulation of this miRNA has suppressed cell proliferation and stimulated the differentiation of keratinocytes. GRHL2 has been recognized as a direct target of miR-194 [30]. The STAT3 suppressor miRNA, miR-4516 has been down-regulated in skin samples obtained from patients with psoriasis. This miRNA also targets the extracellular matrix protein fibronectin 1 and integrin subunit α9. Forced over-expression of miR-4516 in human keratinocytes has been shown to inhibit cell motility and proliferation through inhibition of expression of genes establishing cytoskeletal reorganization. In addition, it stimulates terminal differentiation of these cells [31].

Table 4 shows the list of miRNAs whose expression has been down-regulated in the psoriasis.

**Table 4:** List of miRNAs whose expression has been down-regulated in the psoriasis.

miRNA	Numbers of clinical samples	Source	Targets/Regulators	Signaling Pathways	Function and comments	Reference
miR-125b	25 psoriasis patients and 27 normal individuals were recruited.	Keratinocytes	FGFR2	Cell proliferation and differentiation	Overexpression of miR-125b in primary human keratinocytes repressed proliferation and induced the expression of several known differentiation markers.	[25]
	A total of 32 psoriasis subjects and 10 healthy volunteers were enrolled in the present study.	Serum, HaCaT and 293T cells	BRD4	Jagged-1/Notch signaling pathway	miR-125b tightly binds to BRD4 and confines the translation process of the Jagged-1 ligand. By suppressing the activation of the Notch signaling pathway, miR-125b inhibits the proliferation of psoriasis cells.	[26]
let-7b	4 patients with psoriasis and 4 healthy subjects were enrolled.	Keratinocytes	IL-6	ERK1/2 signaling pathway	Let-7b directly targets IL-6, an indispensable cytokine regulating cell differentiation, which is induced in the affected epidermis of psoriasis patients.	[27]
miR-145-5p	10 patients with psoriasis and 10 healthy subjects were recruited in this study.	Keratinocytes	MLK3, STAT3 and NF-ҡB	AKT/GSK, MAPK, mTOR and NF-κB signaling pathways	Overexpression of miR-145-5p in normal human epidermal keratinocytes inhibited cell proliferation and production of chemokines. Silencing miR-145-5p enhanced NHEK proliferation and augmented chemokine secretion.	[28]
miR-187	Psoriatic skin samples and adjacent uninvolved psoriatic skin samples were obtained.	Keratinocytes	CD276	CD276‐STAT3 signaling pathways	Overexpression of miR‐187 reduced keratinocytes hyperproliferation.	[29]
miR-194	15 patients with psoriasis and 10 healthy subjects were enrolled in the present study	Keratinocytes	GRHL2		Overexpression of miR-194 repressed the proliferation and stimulated the differentiation of primary human keratinocytes, whereas miR-194 suppression stimulated the proliferation and repressed their differentiation.	[30]
miR-4516	Biopsies from lesional (n = 15) and non-lesional (n = 3) area were obtained from psoriasis patients. Foreskin samples served as healthy controls.	Keratinocytes	FN1, ITGA9, STAT3, Bcl xl and Cyclin D1		miR-4516 silencing in psoriatic skin might contribute in enhanced migration, resistance to apoptosis and differentiation as seen in psoriasis lesional keratinocytes.	[31]
miR-876-5p	Psoriasis and normal skin tissues were collected from 10 psoriasis patients.	Keratinocytes/plasma	Ang-1	PI3K/AKT and ERK signaling pathway	Invasion and adhesion, serving as important behavioral traits of epidermal keratinocytes cells, were suppressed by excessive miR-876-5p in psoriasis cells	[32]
miR-181b-5p	35 cases of psoriatic lesional skin tissues and 25 cases of healthy skin tissues were collected.	Human epidermal keratinocytes (HEKs)	Akt3	Akt/mTOR Signaling	Upregulation of miR-181b-5p inhibited HEKs proliferation at least partly by targeting Akt3.	[33]
miR‐181b	28 patients diagnosed with psoriasis and 20 healthy controls who underwent plastic surgery were recruited.	Human epidermal keratinocytes (HEKs)	TLR4	TLR4 pathway	miR‐181b negatively regulates the proliferation of HEKs in psoriasis and might provide new insights for seeking novel targets of treatment and prognosis of psoriasis.	[11]
miR-486-3p	Psoriatic lesions and the adjacent non-lesional skin were collected from 25 patients. Normal skin harvested from 25 healthy Participants.	Epidermis	K17	TGFβ/SMAD/miR-486-3p Signaling	Downregulated miR-486-3p, allowed over-expression of K17, driving keratinocyte proliferation, and thus contributes to the development of psoriasis.	[34]
miR-126	147 psoriasis patients and 120 healthy volunteers were enrolled in this clinical study.	Plasma			miR-126 plays a positive role in the inhibition of inflammation, and its low concentration may allow a greater influx of inflammatory cells to enter the skin, further aggravating inflammation in psoriasis patients.	[35]
miR-143	In total, 194 psoriasis patients and 175 healthy controls were recruited.	Skin tissues/PBMCs	Bcl-2		miR-143 expression in PBMCs is negatively correlates with disease severity in psoriasis and thus a low-expression of miR-143 in PBMCs would indicate poor prognosis for this disease.	[36]
miR-424	Skin specimens were obtained from 6 psoriasis patients and 6 healthy controls. Serum samples were taken from 15 patients with psoriasis and sex- and age- matched healthy volunteers.	Skin tissue/serum	MEK1	miR-424 ⁄MEK1⁄ cyclin E1 pathway	Inhibiting miR-424 in normal human keratinocytes led to upregulation of MEK1 or cyclin E1 protein, and resulted in increased cell proliferation.	[37]
miR-138	40 patients with psoriasis and 35 healthy subjects were included in this study.	CD4(+) T cells	RUNX3		Overexpression of miR-138 inhibits RUNX3 expression and decreased the ratio of Th1/Th2 in CD4(+) T cells.	[38]

## 5 Up-regulated miRNAs in psoriasis

The miRNA miR-31 has been shown to be induced by NF-κB in psoriatic epidermis. Knock out of this miRNA suppresses keratinocytes hyperproliferation, reduces acanthosis and diminishes the disease severity in the animal models of psoriasis. miR-31 has functional interaction with ppp6c to inhibit its expression [39]. This miRNA has also been shown to directly bind with the serine/threonine kinase 40 (STK40), a negative regulator of NF-κB signaling to inhibit its expression. Expression of this miRNA is increased by TGF-β1, a cytokine which is over-expressed in psoriasis skin [40]. STK40 has also been targeted by miR-130a, a miRNA that enhances viability and migration of keratinocytes. In addition, miR-130a indirectly regulates SOX9-associated JNK/MAPK signaling [41]. Expression of miR-126 has been increased in psoriatic lesions compared with paired normal skin samples in association with Psoriasis Area and Severity Index (PASI) score. Forced over-expression of miR-126 has increased cell proliferation and reduced apoptosis in HaCaT cells. These effects were accompanied by over-production of TNF-α, IFN-γ, IL-17A, and IL-22 while down-regulation of IL-10 levels [42]. In addition, expression levels of miR-146a and miR-146b have been increased in the psoriatic lesions. Proinflammatory cytokines have a prominent role in induction of expression of these miRNAs particularly miR-146a in keratinocytes and fibroblasts. FERMT1 has been identified as a target of miR-146a. Notably, reassessment of genome-wide data from of a large cohort of psoriasis patients and healthy subjects revealed a moderate association between genetic variants in the miR-146a coding gene and this disorder. These two miRNAs have been suggested to alter inflammatory reaction and proliferation of keratinocyte [43]. Expression of miR-142–3p has been shown to be elevated in M5-induced HaCaT cells. Suppression of miR-142–3p significantly inhibited proliferation and increased apoptosis in these cells, as demonstrated by diminished levels of the antiapoptotic Bcl-2 protein, and simultaneous enhancement of the proapoptotic protein Bax. These events were accompanied by reduction of M5-associated inflammatory reactions. Notably, miR-142–3p has been shown to directly target Sema3A. Therefore, suppression of miR-142–3p defend HaCaT cells against M5-associated hyperproliferation and inflammation-induced damage by modulation of expression of its target, indicating the role miR-142–3p/Sema3A axis as a therapeutic target for precluding keratinocyte [44]. Expression of miR-155 has been shown to be elevated in psoriasis tissues compared with normal tissues [45] and in peripheral blood mononuclear cells of these patients compared with controls [46]. miR-155 silencing significantly diminished cell proliferation, altered cell cycle progression and enhanced the quantity of apoptotic cells. Moreover, its silencing modulated expression of several apoptosis-associated genes including PTEN, AKT, Bax and Bcl-2 [45]. Over-expression of miR-21 has been demonstrated in skin abrasions of patients with psoriasis and in association with down-regulation of TIMP-3 expression and stimulation of TACE/ADAM17. In vitro and in vivo studies have shown that over-expression of miR-21 is resulted from dysregulation of activity of Jun/AP-1 and subsequent induction of the IL-6/STAT3 axis. Suppression of miR-21 amended pathological features both in xenotransplants and in a psoriasis-like mouse model indicating the role of this miRNA as a therapeutic target in psoriasis [47]. Expression of miR-200s has been shown to be higher in affected skin of patients with psoriasis compared with both their unaffected skin samples and skin samples from controls. Moreover, expressions of miR-200c and miR-200a were higher in peripheral blood of patients compared with controls. There were significant correlations between expression levels of miR-200c and both PASI and disease duration as well as determinants of heart dysfunction [48]. miR-210 is another up-regulated miRNA in the psoriasis. This miRNA has been shown to stimulate Th17 and Th1 cell differentiation but preclude Th2 differentiation via suppressing STAT6 and LYN expression. Suppression of miR-210 expression has reversed the immune imbalance and the progress of psoriasis-like inflammatory responses animal models of psoriasis. Mechanistically, expression of this miRNA is induced by TGF-β and IL-23 through activation of HIF-1α [49]. Expression of miR-210 has also been up-regulated in CD4(+) T cells of patients with psoriasis vulgaris. This miRNA negatively regulates expression of FOXP3, thus weakening the immunosuppressive effects of Treg cells in CD4(+) T cells [50]. Expression levels of miR-369-3p, miR-1266 and miR-31 have been elevated in serum samples of patients with psoriasis [[51], [52], [53]], indicating a possible role for these miRNAs as non-invasive diagnostic markers for this skin disorder. Table 5 shows the list of up-regulated miRNAs in the psoriasis.

**Table 5:** List of miRNAs whose expression has been up-regulated in the psoriasis.

miRNA	Numbers of clinical samples (tissues, serum, etc.)	Source	Targets/Regulators	Signaling Pathways	Function and comments	Reference
miR-31	29 Psoriatic skin samples were obtained by punch biopsy and normal adult human skin specimens were taken from healthy adults undergoing plastic surgery.	Keratinocytes	Ppp6c	NF-κB signaling pathway	Ppp6c is directly targeted by miR-31 and its silencing led to an increase in the epidermis thickness and an enhanced proliferation of keratinocytes	[39]
	Biopsies were taken from nonlesional (n = 20) and lesional skin (n = 43) of patients with psoriasis and from skin of healthy individuals (n = 35).	Keratinocytes	STK40	NF-κB signaling	Overexpression of miR-31 contributes to skin inflammation in psoriasis lesions by regulating the production of inflammatory mediators and leukocyte chemotaxis to the skin.	[40]
miR-130	Specimens were collected from 12 patients with psoriasis and 8 healthy controls.	Keratinocytes	STK40, NF-κB p65, SOX9, p-c-Jun, p-JNK, and p-p38MAPK	NF-κB and JNK/MAPK signaling pathways	Overexpressing miR-130a strikingly promoted HaCaT cell viability and migration and inhibited apoptosis	[41]
miR-17-92	Lesional and perilesional specimens from 25 psoriasis patients and healthy skin specimens from 25 people who accepted cosmetic surgery were collected.	Keratinocytes	CDKN2B, SOCS1	STAT1 signaling pathway	miR-17-92 cluster enhances the proliferation and the cell-cycle progression of keratinocytes and facilitates the secretion of CXCL9 and CXCL10 from keratinocytes.	[10]
miR-126	Lesional and paired non-lesional skin were collected from 102 psoriasis patients.	Keratinocytes	C‐caspase, Bcl‐2, TNF‐α, IFN‐γ, IL‐17A and IL‐22	Apoptosis	Upregulation of miR‐126 promotes cells proliferation and inflammation while prevents cells apoptosis in keratinocytes.	[42]
miR-146a/b	30 patients with psoriasis and 30 control subjects were included in the study.	Human epidermal keratinocytes (HEKs)	FERMT1, IRAK1, CCL5, IL-8, CARD10 and NUMB	NF-κB signaling pathway and regulation of cell proliferation	The ability of miR-146a/b to hinder inflammatory responses, activation-induced cell death and proliferation of keratinocytes and fibroblasts proposes that miR-146a/b participate in the skin homeostasis and controlling inflammatory responses in both healthy and diseased skin.	[43]
miR-142-3p	Human keratinocytes HaCaT cells were obtained from the American Type Culture Collection. M5, a cocktail of cytokines, was used to induce psoriatic inflammation like condition in HaCaT cells.	HaCaT cells	Sema3A	miR-142-3p/Sema3A signaling	Suppression of miR-142-3p protects HaCaT cells against M5-induced hyper-proliferation and inflammatory injury by suppressing its target Sema3A.	[44]
miR-155	20 psoriasis tissues and the adjacent normal tissues were analyzed.	Skin Samples	PTEN, PIP3, AKT, p-AKT, Bax and Bcl-2	PTEN and AKT signaling pathway	Down-regulation of miR-155 significantly inhibits proliferation, migration, invasion and promotes apoptosis.	[45]
	11 patients with psoriasis and 11 healthy age- and gender-matched volunteers participated in this study.	PBMCs	SOCS1, VDR	TLR- and IFN-signaling	Psoriasis patients presented increased expression of miR-155 in PBMCs that was correlated with Psoriasis Area Severity Index (PASI) and decreased with disease remission.	[46]
miR-21	32 biopsies (lesional and non-lesional tissues) were obtained from patients with psoriasis.	Skin Samples	TIMP-3, TACE and ADAM17	IL-6/Stat3 pathway	Blocking miR-21 and its target TIMP-3 may be a potential therapeutic strategy for treating psoriasis.	[47]
miR-200c	29 patients with psoriasis and 29 control subjects were recruited.	Skin Samples/plasma	SIRT1, eNOS and FOXO1		miR-200c correlates with the severity of disease and chronic inflammation.	[48]
miR-223	20 patients with psoriasis and 15 healthy volunteers were recruited.	Skin samples/PBMCs/HaCaT cells	PTEN	PTEN/Akt pathway	miR-223 increased proliferation and inhibited apoptosis of IL-22-stimulated keratinocytes.	[54]
miR-210	A total of 63 psoriatic patients and 80 sex- and age-matched healthy controls were recruited.	Skin lesions/PBMCs	STAT6 and LYN	TGF-β/IL-23–HIF-1α–miR-210–STAT6/LYN pathways	Elevated miR-210 expression might participate in the CD4^+^ T cell–mediated immune dysfunction in peripheral and skin lesions of psoriasis.	[49]
	18 patients with psoriasis and 18 age- and sex-matched healthy subjects were enrolled in this study.	CD4(+) T cells	FOXP3, IFN-γ, IL-17, IL-10 and TGF-β		Suppression of miR-210 increases FOXP3 expression and reverses the immune dysfunction in CD4^+^ T cells from patients with psoriasis.	[50]
miR-200a	A total of 189 patients with psoriasis and 109 healthy individuals were recruited.	CD4(+) T cells	RORγt, FOXP3		miR-200a can change the concentrations of Th17 and Treg cells in the peripheral blood of psoriatic patients.	[55]
miR-369-3p	40 patients with psoriasis and 40 healthy subjects were recruited in this study (serum samples were obtained from 30 and skin tissues were collected from 10 individuals in each group).	Skin samples/Serum	TNF, LIMK1, SIRT1, SP3, ADAM10, HES1 and WNT5A		miR-369-3p is a possible biomarker for psoriasis that can appraise the prognosis of psoriasis and may contribute to the development of new therapeutic methods.	[51]
miR-1266	Samples were obtained from 25 patients with psoriasis and 20 healthy age- and sex-matched volunteers.	Serum	IL-17A		Serum miR-1266 may have potential for a new disease marker.	[52]
miR-31	29 patients with psoriasis and 22 healthy blood donors were recruited.	Serum		Apoptosis	miR-31 and ET-1 may serve as potential biomarkers of the disease. ET-1 is made by psoriatic keratinocytes and inhibits apoptosis. Inflammation increases the production of ET-1, which in turn results in the chronic induction of keratinocyte proliferation.	[53]
miR-19a	18 patients with psoriasis and 22 healthy controls were recruited.	Hair root	TNF-α		There is a significant correlation between relative hair miR-19a levels and disease duration. The hair root miR-19a levels can be a marker reflecting the subjective severity of symptoms in psoriasis.	[56]

Altered expression of miRNAs can be used as a tool for differentiation of psoriatic lesions from normal skin samples. Expression levels of miR‐155, let‐7i, miR‐146a, miR‐21 and miR‐223 has been increased in the peripheral blood mononuclear cells of patients with psoriasis. Moreover, expressions of miR‐21, miR‐146a and miR‐223 have been elevated in plasma samples of patients as well. Based on the results of receiver‐operator characteristic (ROC) curve analysis these miRNAs can be used for discriminating patients from healthy subjects. Notably, there was a significant correlation between baseline expression levels of miR-155 in peripheral blood mononuclear cells and PASI score. Expression of this miRNA could differentiate patients' specimens at baseline and after treatment [46]. Table 6 summarizes the results of studies which assessed diagnostic power of miRNAs in the psoriasis.

**Table 6:** Diagnostic value of miRNAs in psoriasis patients.

miRNA	Numbers of clinical samples	Distinguish between	Area under curve (AUC)	Sensitivity	Specificity	Reference
miR-126	147 psoriasis patients and 120 healthy volunteers were enrolled in this clinical study.	Distinguishes psoriasis patients from normal subjects.	0.771	57.1%	87.5%	[35]
miR-143	In total, 194 psoriasis patients and 175 healthy controls were recruited.	Distinguishes psoriasis patients from normal subjects.	0.886	78.5%	97.1%	[36]
		Distinguishes progressive stage	0.884	75.5%	90.3%	
		Distinguishes stable stage	0.833	60.9%	97.1%	
miR-19a	18 patients with psoriasis and 22 healthy controls were recruited.	Distinguishes psoriasis patients from normal subjects.	0.87			[56]
miR-155	11 patients with psoriasis and 11 healthy age- and gender-matched volunteers participated in this study.	Distinguishes psoriasis patients from normal subjects.	0.955	0.91	0.9	[46]

## 6 Circular RNAs (circRNA) and psoriasis

Moldovan et al. have identified circRNA signature of affected and non-affected psoriasis skin using RNA-seq and RNA chromogenic in situ hybridization techniques. They reported a significant decreased in the expression circRNA in affected skin compared to non-affected skin. Totally, they detected 298 unique high-abundance circRNAs in the affected and unaffected skin samples, more than one-third of them being common between these two set of samples. Yet, all of these circRNAs were mostly expressed at lower amounts in the affected tissues. The same situation was reported when comparing the affected and unaffected samples from each individual patient. This expression change has been occurred mostly in the epidermis. Notably, most of circRNAs were decreased independently of their associated linear host genes. Factors that influence circRNA biogenesis or the amounts inflammatory cells in the lesion did not affect the levels of circRNAs. Down-regulation of circRNAs might be due to high proliferation and turnover rates of the keratinocytes in the affected skin and lower differentiation status of these cells [57], since expressions of circRNAs are mostly increased during differentiation [58].

## 7 Conclusions

In the present manuscript, we summarized the data pointing to the dysregulation of lncRNAs and miRNAs in the psoriasis. LncRNAs and miRNAs have functional interactions that are involved in the regulation of immune responses and the pathophysiology of inflammatory disorders such as psoriasis. Massive dysregulation of ncRNAs in the psoriatic lesions indicates that the combined functions of numerous ncRNAs might participate in the pathogenesis of psoriasis [12]. Dysregulated ncRNAs were mostly associated with Treg cells differentiation and activity as well as a number of signaling pathways such as NF-κΒ, mTOR, MAPK and JAK-STAT [12]. Moreover, expressions of several lncRNAs in the keratinocytes have been shown to be modulated by cytokine treatment [13]. Thus, these transcripts are actively involved in the pathophysiology of psoriasis. LncRNAs participate in the pathogenesis of psoriasis through various mechanisms among them is their function as competing endogenous RNAs (ceRNAs) to decrease bioavailability of miRNAs. Several such examples have been identified in the context of psoriasis. For instance, MEG3/miR-21 axis contribute in the pathophysiology of psoriasis through modulation of caspase-8, cleaved caspase-8, cytc, and apaf-1 [14]. Moreover, expression studies in IL-22-stimulated keratinocytes have shown the role of the MSX2P1-miR-6731-5p axis in the regulation of S100A7 [19]. Such lncRNA/miRNA/mRNA trios are putative therapeutic targets in psoriasis.

Genomic variants within lncRNA and miRNA coding genes are expected to alter risk of psoriasis. Yet, few studies have addressed this point. Although associations between HOTAIR and ANRIL genomic variants and risk of psoriasis have been pointed in the Iranian population, the molecular mechanism of such associations has not been identified. Moreover, such associations have not been verified in other populations. Therefore, future investigations should unravel weather these SNPs alter immune responses or proliferation/apoptosis of keratinocytes.

Another subgroup of ncRNAs namely circRNAs have also been shown to be decreased in the psoriatic lesions. Yet, it is not clear whether this altered expression is the cause or effect of psoriasis. There is no evidence regarding the functional role for these transcripts in the pathogenesis of psoriasis through modulation of miRNA profile [57]. Therefore, future studies should focus on the importance of these transcripts in the pathogenesis of this disorder to find whether circRNAs can be used as biomarkers for psoriasis.

Finally, expression levels of miRNAs in the serum or peripheral blood mononuclear cells can distinguish patients from healthy subjects. However, little is known about the diagnostic role of lncRNAs in psoriasis. Based on the functional interaction between miRNAs and lncRNAs, it is expected that some panels of lncRNAs and miRNAs could be used as diagnostic panels for psoriasis. However, this research field should be explored in future.

## References

[1] Trojacka E., Zaleska M., Galus R. (2015). Influence of exogenous and endogenous factors on the course of psoriasis. Pol. Merkur. Lek..

[2] Ferenczi K., Burack L., Pope M., Krueger J., Austin L. (2000). CD69, HLA-DR and the IL-2R identify persistently activated T cells in psoriasis vulgaris lesional skin: blood and skin comparisons by flow cytometry. J. Autoimmun..

[3] Bos J., Hagenaars C., Das P., Krieg S., Voorn W., Kapsenberg M. (1989). Predominance of “memory” T cells (CD4+, CDw29+) over “naive” T cells (CD4+, CD45R+) in both normal and diseased human skin. Arch. Dermatol. Res..

[4] Vollmer S., Menssen A., Prinz J.C. (2001). Dominant lesional T cell receptor rearrangements persist in relapsing psoriasis but are absent from nonlesional skin: evidence for a stable antigen-specific pathogenic T cell response in psoriasis vulgaris. J. Invest. Dermatol..

[5] Boyman O., Hefti H.P., Conrad C., Nickoloff B.J., Suter M., Nestle F.O. (2004). Spontaneous development of psoriasis in a new animal model shows an essential role for resident T cells and tumor necrosis factor-α. J. Exp. Med..

[6] Kamiya K., Kishimoto M., Sugai J., Komine M., Ohtsuki M. (2019). Risk factors for the development of psoriasis. Int. J. Mol. Sci..

[7] Yan J., Song J., Qiao M., Zhao X., Li R., Jiao J. (2019). Long noncoding RNA expression profile and functional analysis in psoriasis. Mol. Med. Rep..

[8] Hadjicharalambous M.R., Lindsay M.A. (2019). Long non-coding RNAs and the innate immune response. Noncoding RNA.

[9] Tsitsiou E., Lindsay M.A. (2009). microRNAs and the immune response. Curr. Opin. Pharmacol..

[10] Zhang W., Yi X., An Y., Guo S., Li S., Song P. (2018 May 1). MicroRNA-17-92 cluster promotes the proliferation and the chemokine production of keratinocytes: implication for the pathogenesis of psoriasis. Cell Death Dis..

[11] Feng C., Bai M., Yu N.Z., Wang X.J., Liu Z. (2017 Feb). MicroRNA-181b negatively regulates the proliferation of human epidermal keratinocytes in psoriasis through targeting TLR4. J. Cell Mol. Med..

[12] Yan J., Song J., Qiao M., Zhao X., Li R., Jiao J. (2019 May). Long noncoding RNA expression profile and functional analysis in psoriasis. Mol. Med. Rep..

[13] Tsoi L.C., Iyer M.K., Stuart P.E., Swindell W.R., Gudjonsson J.E., Tejasvi T. (2015). Analysis of long non-coding RNAs highlights tissue-specific expression patterns and epigenetic profiles in normal and psoriatic skin. Genome Biol..

[14] Jia H.Y., Zhang K., Lu W.J., Xu G.W., Zhang J.F., Tang Z.L. (2019 Oct 28). LncRNA MEG3 influences the proliferation and apoptosis of psoriasis epidermal cells by targeting miR-21/caspase-8. BMC Mol. Cell Biol..

[15] Li H., Yang C., Zhang J., Zhong W., Zhu L., Chen Y. (2020 Mar 3). Identification of potential key mRNAs and LncRNAs for psoriasis by bioinformatic analysis using weighted gene co-expression network analysis. Mol. Genet. Genom.: MGG.

[16] Szegedi K., Sonkoly E., Nagy N., Nemeth I.B., Bata-Csorgo Z., Kemeny L. (2010 Mar). The anti-apoptotic protein G1P3 is overexpressed in psoriasis and regulated by the non-coding RNA, PRINS. Exp. Dermatol..

[17] Gao J., Chen F., Hua M., Guo J., Nong Y., Tang Q. (2018 Sep 4). Knockdown of lncRNA MIR31HG inhibits cell proliferation in human HaCaT keratinocytes. Biol. Res..

[18] Duan Q., Wang G., Wang M., Chen C., Zhang M., Liu M. (2020 Feb 17). LncRNA RP6-65G23.1 accelerates proliferation and inhibits apoptosis via p-ERK1/2/p-AKT signaling pathway on keratinocytes. J. Cell. Biochem..

[19] Qiao M., Li R., Zhao X., Yan J., Sun Q. (2018 Feb 15). Up-regulated lncRNA-MSX2P1 promotes the growth of IL-22-stimulated keratinocytes by inhibiting miR-6731-5p and activating S100A7. Exp. Cell Res..

[20] Rakhshan A., Zarrinpour N., Moradi A., Ahadi M., Omrani M.D., Ghafouri-Fard S. (2020 Jan). Genetic variants within ANRIL (antisense non coding RNA in the INK4 locus) are associated with risk of psoriasis. Int. Immunopharm..

[21] Rakhshan A., Zarrinpour N., Moradi A., Ahadi M., Omrani M.D., Ghafouri-Fard S. (2020 Feb 23). A single nucleotide polymorphism within HOX Transcript Antisense RNA (HOTAIR) is associated with risk of psoriasis. Int. J. Immunogenet..

[22] Timis T.L., Orasan R.I. (2018). Understanding psoriasis: role of miRNAs. Biomed. Rep..

[23] Fu D., Yu W., Li M., Wang H., Liu D., Song X. (2015). MicroRNA-138 regulates the balance of Th1/Th2 via targeting RUNX3 in psoriasis. Immunol. Lett..

[24] Zhao M., Wang L-t, Liang G-p, Zhang P., Deng X-j, Tang Q. (2014). Up-regulation of microRNA-210 induces immune dysfunction via targeting FOXP3 in CD4+ T cells of psoriasis vulgaris. Clin. Immunol..

[25] Xu N., Brodin P., Wei T., Meisgen F., Eidsmo L., Nagy N. (2011 Jul). MiR-125b, a microRNA downregulated in psoriasis, modulates keratinocyte proliferation by targeting FGFR2. J. Invest. Dermatol..

[26] Pan M., Huang Y., Zhu X., Lin X., Luo D. (2019 Jun). miR125bmediated regulation of cell proliferation through the Jagged1/Notch signaling pathway by inhibiting BRD4 expression in psoriasis. Mol. Med. Rep..

[27] Wu Y., Liu L., Bian C., Diao Q., Nisar M.F., Jiang X. (2018 Sep 15). MicroRNA let-7b inhibits keratinocyte differentiation by targeting IL-6 mediated ERK signaling in psoriasis. Cell Commun. Signal. : CCS.

[28] Yan J.J., Qiao M., Li R.H., Zhao X.T., Wang X.Y., Sun Q. (2019 Feb). Downregulation of miR-145-5p contributes to hyperproliferation of keratinocytes and skin inflammation in psoriasis. Br. J. Dermatol..

[29] Tang L., He S., Zhu Y., Feng B., Su Z., Liu B. (2019 Apr). Downregulated miR-187 contributes to the keratinocytes hyperproliferation in psoriasis. J. Cell. Physiol..

[30] Yu X., An J., Hua Y., Li Z., Yan N., Fan W. (2017 Feb). MicroRNA-194 regulates keratinocyte proliferation and differentiation by targeting Grainyhead-like 2 in psoriasis. Pathol. Res. Pract..

[31] Chowdhari S., Sardana K., Saini N. (2017 Dec). miR-4516, a microRNA downregulated in psoriasis inhibits keratinocyte motility by targeting fibronectin/integrin alpha9 signaling. Biochim. Biophys. Acta (BBA) - Mol. Basis Dis..

[32] Rongna A., Yu P., Hao S., Li Y. (2018 Jul). MiR-876-5p suppresses cell proliferation by targeting Angiopoietin-1 in the psoriasis. Biomed. Pharmacother..

[33] Zheng Y., Cai B., Li X., Li D., Yin G. (2019 Nov 5). MiR-125b-5p and miR-181b-5p inhibit keratinocyte proliferation in skin by targeting Akt3. Eur. J. Pharmacol..

[34] Jiang M., Sun Z., Dang E., Li B., Fang H., Li J. (2017 Oct). TGFbeta/SMAD/microRNA-486-3p signaling Axis mediates Keratin 17 expression and keratinocyte hyperproliferation in psoriasis. J. Invest. Dermatol..

[35] Duan Y., Zou J., Mao J., Guo D., Wu M., Xu N. (2019 Jul). Plasma miR-126 expression correlates with risk and severity of psoriasis and its high level at baseline predicts worse response to Tripterygium wilfordii Hook F in combination with acitretin. Biomed. Pharmacother..

[36] Zheng Y.Z., Chen C.F., Jia L.Y., Yu T.G., Sun J., Wang X.Y. (2017 Aug 1). Correlation between microRNA-143 in peripheral blood mononuclear cells and disease severity in patients with psoriasis vulgaris. Oncotarget.

[37] Ichihara A., Jinnin M., Yamane K., Fujisawa A., Sakai K., Masuguchi S. (2011 Nov). microRNA-mediated keratinocyte hyperproliferation in psoriasis vulgaris. Br. J. Dermatol..

[38] Fu D., Yu W., Li M., Wang H., Liu D., Song X. (2015 Jul). MicroRNA-138 regulates the balance of Th1/Th2 via targeting RUNX3 in psoriasis. Immunol. Lett..

[39] Yan S., Xu Z., Lou F., Zhang L., Ke F., Bai J. (2015 Jul 3). NF-kappaB-induced microRNA-31 promotes epidermal hyperplasia by repressing protein phosphatase 6 in psoriasis. Nat. Commun..

[40] Xu N., Meisgen F., Butler L.M., Han G., Wang X.J., Soderberg-Naucler C. (2013 Jan 15). MicroRNA-31 is overexpressed in psoriasis and modulates inflammatory cytokine and chemokine production in keratinocytes via targeting serine/threonine kinase 40. J. Immunol..

[41] Xiong Y., Chen H., Liu L., Lu L., Wang Z., Tian F. (2017 Mar). microRNA-130a promotes human keratinocyte viability and migration and inhibits apoptosis through direct regulation of STK40-mediated NF-kappaB pathway and indirect regulation of SOX9-meditated JNK/MAPK pathway: a potential role in psoriasis. DNA Cell Biol..

[42] Feng S., Wang L., Liu W., Zhong Y., Xu S. (2018 Nov). MiR-126 correlates with increased disease severity and promotes keratinocytes proliferation and inflammation while suppresses cells' apoptosis in psoriasis. J. Clin. Lab. Anal..

[43] Hermann H., Runnel T., Aab A., Baurecht H., Rodriguez E., Magilnick N. (2017 Sep). miR-146b probably assists miRNA-146a in the suppression of keratinocyte proliferation and inflammatory responses in psoriasis. J. Invest. Dermatol..

[44] Zhang D., Wang Y., Xia Y., Huo J., Zhang Y., Yang P. (2020 Apr). Repression of miR-142-3p alleviates psoriasis-like inflammation by repressing proliferation and promoting apoptosis of keratinocytes via targeting Sema3A. Mol. Cell. Probes.

[45] Xu L., Leng H., Shi X., Ji J., Fu J., Leng H. (2017 Jun). MiR-155 promotes cell proliferation and inhibits apoptosis by PTEN signaling pathway in the psoriasis. Biomed. Pharmacother..

[46] Garcia-Rodriguez S., Arias-Santiago S., Blasco-Morente G., Orgaz-Molina J., Rosal-Vela A., Navarro P. (2017 Feb). Increased expression of microRNA-155 in peripheral blood mononuclear cells from psoriasis patients is related to disease activity. J. Eur. Acad. Dermatol. Venereol. : JEADV.

[47] Guinea-Viniegra J., Jimenez M., Schonthaler H.B., Navarro R., Delgado Y., Concha-Garzon M.J. (2014 Feb 26). Targeting miR-21 to treat psoriasis. Sci. Transl. Med..

[48] Magenta A., D'Agostino M., Sileno S., Di Vito L., Uras C., Abeni D. (2019). The oxidative stress-induced miR-200c is upregulated in psoriasis and correlates with disease severity and determinants of cardiovascular risk. Oxid. Med. Cell Longev..

[49] Wu R., Zeng J., Yuan J., Deng X., Huang Y., Chen L. (2018 Jun 1). MicroRNA-210 overexpression promotes psoriasis-like inflammation by inducing Th1 and Th17 cell differentiation. J. Clin. Invest..

[50] Zhao M., Wang L.T., Liang G.P., Zhang P., Deng X.J., Tang Q. (2014 Jan). Up-regulation of microRNA-210 induces immune dysfunction via targeting FOXP3 in CD4(+) T cells of psoriasis vulgaris. Clin. Immunol..

[51] Guo S., Zhang W., Wei C., Wang L., Zhu G., Shi Q. (2013 Sep-Oct). Serum and skin levels of miR-369-3p in patients with psoriasis and their correlation with disease severity. Eur. J. Dermatol. : EJD.

[52] Ichihara A., Jinnin M., Oyama R., Yamane K., Fujisawa A., Sakai K. (2012 Jan-Feb). Increased serum levels of miR-1266 in patients with psoriasis vulgaris. Eur. J. Dermatol. : EJD.

[53] Borska L., Andrys C., Chmelarova M., Kovarikova H., Krejsek J., Hamakova K. (2017 Dec 20). Roles of miR-31 and endothelin-1 in psoriasis vulgaris: pathophysiological functions and potential biomarkers. Physiol. Res..

[54] Wang R., Wang F.F., Cao H.W., Yang J.Y. (2019 Aug 1). MiR-223 regulates proliferation and apoptosis of IL-22-stimulated HaCat human keratinocyte cell lines via the PTEN/Akt pathway. Life Sci..

[55] Wang X.Y., Chen X.Y., Li J., Zhang H.Y., Liu J., Sun L.D. (2017 Sep). MiR-200a expression in CD4+ T cells correlates with the expression of Th17/Treg cells and relevant cytokines in psoriasis vulgaris: a case control study. Biomed. Pharmacother..

[56] Hirao H., Jinnin M., Ichihara A., Fujisawa A., Makino K., Kajihara I. (2013 Nov-Dec). Detection of hair root miR-19a as a novel diagnostic marker for psoriasis. Eur. J. Dermatol. : EJD.

[57] Moldovan L.I., Hansen T.B., Veno M.T., Okholm T.L.H., Andersen T.L., Hager H. (2019 Nov 27). High-throughput RNA sequencing from paired lesional- and non-lesional skin reveals major alterations in the psoriasis circRNAome. BMC Med. Genom..

[58] Rybak-Wolf A., Stottmeister C., Glažar P., Jens M., Pino N., Giusti S. (2015). Circular RNAs in the mammalian brain are highly abundant, conserved, and dynamically expressed. Mol. Cell.

